# Coping strategies employed by public psychiatric healthcare workers during the COVID-19 pandemic in southern Gauteng, South Africa

**DOI:** 10.1371/journal.pone.0277392

**Published:** 2023-08-10

**Authors:** Ann Scheunemann, Andrew W. Kim, Aneesa Moolla, Ugasvaree Subramaney

**Affiliations:** 1 Health Economics and Epidemiology Research Office, Department of Internal Medicine, School of Clinical Medicine, Faculty of Health Sciences, University of the Witwatersrand, Johannesburg, South Africa; 2 T.H. Chan School of Public Health, Harvard University, Boston, Massachusetts, United States of America; 3 SAMRC Developmental Pathways for Health Research Unit, Faculty of Health Sciences, University of the Witwatersrand, Johannesburg, South Africa; 4 Department of Anthropology, University of California, Berkeley, Berkeley, California, United States of America; 5 Department of Psychiatry, University of the Witwatersrand, Johannesburg, South Africa; St John’s University, UNITED STATES

## Abstract

Within the context of the novel coronavirus pandemic and new challenges to a resource-constrained public healthcare system, many healthcare workers in South Africa have faced numerous stressors that have compromised their mental health. While the current literature on COVID-19 in South Africa highlights the widespread psychosocial stress experienced by healthcare workers during the pandemic, little is known about the coping strategies utilized to continue service delivery and maintain one’s mental health and well-being during this ongoing public health emergency. In this study, we sought to explore the coping strategies used by healthcare workers employed in the public psychiatric care system in southern Gauteng, South Africa during the coronavirus disease (COVID-19) pandemic. Psychiatric healthcare workers (n = 55) employed in three tertiary public hospitals and two specialized psychiatric facilities participated in in-depth interviews between July 2020 and March 2021. We found that coping strategies spanned multi-level and multi-systemic efforts. Intrapersonal, interpersonal, material, and structural coping were mapped across individual, family, and hospital systems. The most commonly utilized coping strategies included positive mindsets and reappraisal, social support systems, and COVID-19 specific protections. Findings also highlighted the contextual and interconnected nature of coping. Healthcare workers applied multiple coping strategies to combat the negative mental health effects of the COVID-19 pandemic. Better understanding these strategies, contexts in which they are employed, and how they interact can be used to develop evidence-based interventions to support healthcare workers experiencing healthcare-related stressors during the COVID-19 pandemic.

## Introduction

In late 2019 the COVID-19 coronavirus emerged, sparking a pandemic which has resulted in hundreds of millions of confirmed cases, millions of deaths [1], and variable social and governmental responses to reduce transmission globally [2]. Fear of the pandemic’s spread prompted a strict government lockdown in South Africa [3], which came to be criticized due to its harsh nature [4, 5] and associations with negative personal, social, and economic outcomes [6–9].

Additionally, the societal effects of the pandemic in South Africa further stressed an already constrained public healthcare system [10] due to a lack of personal protective equipment (PPE), increased patient mortality [11], increased substance use and mental health challenges in healthcare workers [12], and a rise in non-communicable disease risks [8]. Similar to previous pandemics and epidemics, healthcare workers experienced worse symptoms of depression, anxiety, and post-traumatic stress disorder (PTSD) [13, 14], in addition to increased sleep disorders [15], burnout [16], stress, and trauma, all compounding the poor mental health effects resulting from the chronic shortcomings of South Africa’s public healthcare system [10, 12], with potentially worse outcomes for frontline workers [16].

Given this context, successful coping–the process of managing stress and adversity [17]–is crucial to maintaining mental health and general wellbeing among healthcare workers. In situations of complex stress or trauma such as those of the pandemic, individuals tend to employ multiple coping strategies transactionally and flexibly to adapt, based on both intrapersonal and situational characteristics [18] and broadly focused on emotion regulation or taking action to change the situation [19]. Recent research has highlighted limitations of Lazarus’ theory of emotion- and problem-focused coping [19], such as a lack of conceptual clarity, mutual exclusivity, and functional homogeneity [20]. Skinner et al. [20] proposes organizing coping into “families” that are clearly separated by function and higher order adaptive processes–preferences based on situational constraints (e.g., reappraisal), taking action to change the situation (e.g., problem-solving), and utilization of social resources (e.g., self-reliance or social support)–which could improve coping assessment and comparison across studies, and which relate coping to resilience.

Problem- and emotion-focused coping are also not mutually exclusive [20, 21], and conceptualizations like the Coping Circumplex Model have suggested expanding and defining relationships between coping categories [21]. For example, reframing or reappraisal to make meaning through optimism or a positive mindset may also be important when attempting to negotiate traumatic events which cause individuals to grapple with identity and beliefs [22–24]. Social support is also associated with more positive outcomes in traumatic situations, so long as the interpersonal interactions are positive [22]. Other forms of coping include distractions, stress relief, and emotional regulation [20, 21, 25, 26].

Studies on COVID-19 and past healthcare emergencies have shown that healthcare workers have employed coping strategies that span intrapersonal, interpersonal, and situational designations [14, 27]. Work-related coping strategies focus on taking action and using social supports [17]: COVID-19 protective measures, management support, peer support, teamwork, flexibility at work, and appropriate training or knowledge [7, 13–15, 28–30]. Personal coping strategies include family, friend, and community support, acceptance, personal traits such as self-motivation, avoidance strategies, spirituality, and psychological support [7, 13–15, 29–31]. Social support, resilience, and prior experience in health crises provide protection against negative psychological outcomes, including depression, anxiety, distress, insomnia, and somatization [28]. Distraction likewise protects against negative mental health outcomes [15]. Experiential meaning-making may provide benefits beyond protection, supporting post-traumatic growth through a reappraisal of events [16, 22].

Despite limited research in South Africa, studies suggest that training, experience, and preparation related to COVID-19 are associated with greater confidence in COVID-19 knowledge [32] and lower levels of PTSD symptoms [6] in healthcare workers. One small study based in East London found that active coping, planning, and religion were protective factors early in the pandemic. Furthermore, positive framing, active coping, and acceptance were more protective against anxiety, depression, and burnout two months later [33], suggesting that healthcare workers may prioritize coping factors differently at different stages in the pandemic. While these studies suggest that successful coping may be vital, the current literature is limited to a handful of studies and further research is necessary to understand coping among healthcare workers during the COVID-19 pandemic in South Africa. Furthermore, no in-depth qualitative study has examined coping in South African healthcare workers during the pandemic. This study aimed to fill that gap by identifying and examining coping strategies and resources used by public healthcare workers in South Africa during the first two waves of the COVID-19 pandemic, through a content analysis of semi-structured interviews.

## Methods

This study examines perspectives from hospital staff across five major psychiatric facilities in the southern Gauteng region of South Africa. To mitigate infection risk, participants completed interviews telephonically. Interviews were summarized into field notes immediately afterwards, with interviewers adding additional reflections about the participants’ affect, demeanor, and any other non-verbal cues. Codes were developed both inductively and deductively, and two individuals coded the qualitative data. Data were thematically analyzed. The analysis took a descriptive approach to understand perceptions and experiences of coping and stress while being deployed to work during the COVID-19 pandemic due to the novelty of the disease, lockdown, and healthcare environment.

### Study site

This study was conducted with healthcare workers and other hospital-based staff based in the psychiatry departments of three public tertiary hospitals (Charlotte Maxeke Johannesburg Academic Hospital, Chris Hani Baragwanath Academic Hospital, and Helen Joseph Hospital) and two specialized psychiatric hospitals (Sterkfontein Psychiatric Hospital, and Tara Psychiatric Hospital) located in southern Gauteng.

### Recruitment & participant selection

Participants were recruited based on the following criteria. Inclusion criteria included: 18 years and older, worked in one of five psychiatric facilities during the COVID-19 pandemic, and were able to provide informed consent. Exclusion criteria included: individuals who were not able to consent due to perceptual or cognitive disabilities. To reduce infection risk, participants were recruited through online means (e.g. email) and through word-of-mouth, using convenience and snowball sampling methods. Additionally, a minimum of five individuals for each of the following hospital divisions were recruited to gather perspectives from each institution: consultants (i.e. head psychiatrists), staff psychiatrists, allied health workers (e.g. psychologists, occupational therapists, social workers), nurses, administrative staff. Data were collected until data saturation for each of the major research themes was obtained (e.g. stress, coping, perceptions of COVID-19, etc.). A minimum of 24 interviews was needed to obtain meaning saturation [34]. Finally, participants who wished to take part in data collection were not turned away as many hospital staff members requested the opportunity to share their experiences under the lockdown and reflect on the challenges of providing healthcare during the pandemic. A major fire in one of the hospitals required redeploying patients and healthcare staff across the public mental healthcare system in the region, which severely limited the availability of participants and prompted our team to end data collection.

### Data collection

Interviews included a short survey of socio-demographic characteristics, after which participants were asked open-ended questions that assessed experiences with service delivery during the COVID-19 pandemic, sources of stress and coping, and changes in clinical care across the course of the pandemic. Most interviews were conducted telephonically and ranged between 45 minutes to three hours, with a mean of about 1.25 hours. Research assistants administered the interview using the preferred languages of the participant, which included English, isiXhosa, isiZulu. Interviews were conducted between July 2020 and March 2021. The study protocol was approved by the Human Research Ethics Committee at the University of the Witwatersrand, Johannesburg (Clearance number: M190545).

### Data analysis

Interviews were recorded and extensively summarized into field notes which captured key ideas and experiences, and observable behaviors, cues, or interactions. Researchers transcribed and reviewed field notes concurrent to the data collection phase. This process was reflective and systematic, which provided a broader understanding of the data. Researchers then used field notes to develop and discuss key ideas and patterns with qualified qualitative research assistants who were closely connected with the data. Any identified interpretive discrepancies were discussed and resolved at this level. Constantly evaluating and re-evaluating the already grouped data helped the research team to come up with new research questions and observations that informed the next course of data collection to a point where no new themes emerged.

Data from the field notes were coded and extracted using a constructivist research paradigm paired with content analysis [35] and Dedoose 9.0.17 software. Coders both had doctoral degrees in psychology and had focused their training on psychological resilience. Methodological integrity was assured through constant reflexivity and engagement with the literature, as well as through regular discussions about coding patterns, discrepancies, and possible questions. An initial codebook was developed a priori and deductively, based on prior theory and previous publications [12]. Additional codes were added inductively, after an initial read of the data. Coded data were then extracted and thematically analyzed to identify coping strategies.

## Results

Data from interviews with 55 hospital-based workers were organized into categories and themes within broader intrapersonal, interpersonal, structural, and material coping strategies.

### Intrapersonal coping

Intrapersonal factors in coping include any psychological, mental, or emotional factors within an individual which foster or reinforce the ability of the participant to maintain well-being or continue functioning in daily life. We found five themes of intrapersonal coping: self-reliance, mindset and reappraisal, acceptance, manifestations of agency, and refocused attention.

#### Self-reliance

Self-reliance fostered mental fortitude among some healthcare workers. Self-motivation served as a foundation for providing comfort through education and became an alternative to focusing on fear: “Learning more about this disease, how it spread and how you can protect yourself, that was a relief” (42-year-old participant). It helped one participant to confront challenges, leading to positive mental and professional outcomes: “We were forced to learn about COVID-19 and consequently became more confident about the COVID-19 situation. As our experience grew, the decisions became easier to make and we felt less anxiety and fear for ourselves as well as others. I feel that I have gained valuable knowledge and experience through my role in our anti-COVID-19 effort and see it as a positive in my professional development” (48-year-old participant). Self-development and experience were seen to foster resilience, confidence, care, and prioritization, all of which were critical to an effective COVID-19 response. Other participants cited self-reflection and introspection as tools to help them cope: “I think this whole year like with how difficult it’s been has also resulted in a lot of reflection for I think a lot of people just on like what’s important in life. I think because of this year, it’s really caused me to think about the kind of life I want to live… and I think this has influenced a lot of my decisions moving forward… I don’t know if I would have really taken time to make like solid decisions” (31-year-old participant). Additional personal attributes that were protective for a few participants during the pandemic included a sense of humor, compassion, mindfulness, and a sense of justice.

#### Meaning-making: Positive mindset and reappraisal

A positive mindset, or ways an individual cognitively processes and makes sense of an event based on their own beliefs, attitudes, and realities [36], was another way that healthcare workers coped with pandemic-related stressors. Many participants expressed gratitude, or an appreciation for life, health, or work [37], and used appreciation to combat rumination. Some participants were grateful that they had not become ill with COVID-19. One participant who tested positive for COVID-19 still expressed gratitude that she was able to keep her son safe: “My son was in Limpopo during the beginning of lockdown, I had taken everyone out of my house thinking that I may come back with it one day, and I was right… luckily my house was empty” (36-year-old participant). Additionally, given that South Africa’s unemployment rate was high before the pandemic [38] and has worsened over the past year, some healthcare workers expressed gratitude for their continued employment. Other participants found fulfillment or value in promoting the wellbeing of others: “I was scared even to go next to the patient but the following day I made my mind that I’m here to help as a nurse, I’m here to take care so if God allow me to live, I will live” (a middle-aged participant, age unknown). Valuing their vocation protected one participant from feelings of guilt or shame associated with the pandemic: “I actually love my work… I’m a person that likes to think that these other people need more help than I do because I’m physically healthy, and I feel you need to be there for somebody else” (an older participant, age unknown). Another healthcare worker expressed gratitude that her work required her to leave her house during South Africa’s most severe lockdown, which allowed her to go outside and see the sun. Finally, some participants specifically expressed appreciation for the support of colleagues at work.

Healthcare workers felt optimistic and hopeful when reflecting that the pandemic is impermanent or less impactful than they had originally imagined. Some imagined the possibility of life “getting back to normal”, while others predicted a new normal. Other participants felt that government or institutional responses would improve over time and as prevalence rates fell, allowing for better infrastructure protocols, efficiency, preparedness, border reopening, and the ability to resume past activities like traveling. One participant coped by imagining future therapeutic supports for COVID-19: “Well I believe that somewhere, someday, someone is gonna find–not a cure but something to treat this so it’s not gonna be here forever. Okay, it’s gonna be here forever, but it’s not gonna take a lot of lives like it did this year” (23-year-old participant). For some, optimism was tied to an increase in confidence; as they gained understanding and experience around the pandemic, they felt better able to take appropriate precautions, to treat patients who fell ill, to assist in making decisions at work, and to generally adapt.

Spiritual and religious belief systems provided emotional support. Participants prayed, read the bible or participated in online church services to find support, and one participant mentioned reading relevant scriptures more once in-person church services closed: “I’m a Christian, but because we couldn’t go to church, so I relied on prayer and reading the bible and scriptures that were quite motivating–some may sound like exactly the situation that is currently happening. This was another form of support… and hope that it will go away” (41-year-old participant). Some healthcare workers shared their spirituality with their families, by reading scripture and praying together. Spiritual wellness groups were also established at some hospitals to support healthcare workers, and could include therapy, prayer, posting audio messages for those infected, and material support such as groceries.

A small number of participants used reappraisal [19] to cognitively reframe [18] their situations and create new meanings focused on positive changes that resulted from difficult situations, such as greater confidence at work or increased knowledge and confidence in using technology like online meeting platforms. One participant mentioned that the lockdown measures such as the alcohol ban had some benefits, a claim supported by recent data [39]. A few healthcare workers had fewer expenses or had spouses whose business had benefited from the pandemic and were therefore able to save money during lockdown. Finally, one participant found herself motivated by the challenge of the pandemic, which helped to counter any worry.

#### Acceptance

Acceptance allowed participants to not only reckon with the reality of the pandemic but also to actively consider their thoughts and emotions regarding its impacts [40]. One participant coped with the dangers in her workplace by accepting that the virus was ubiquitous and that she was likely to contract it, which helped her to feel less worried at work: “I would be surprised if I didn’t catch it because I work at hospital. Despite the many precautions we take… chances are you’re going to get it. I just knew that somewhere, somehow, I was going to get it. So, I wasn’t really surprised or worried about it, or blaming of anybody because I think it’s one of those things–despite how many of the precautions you take, at some point, you might get it. And if not you, somebody very close to you” (36-year-old participant).

Among those who tested positive for COVID-19, participants coped by accepting their illness. Many participants described getting used to the idea that some changes may be permanent. Even acclimating to the necessity of change was a process, as one healthcare worker noted: “Also important, I feel, is acceptance of the fact that this is new for all of us and that there will be mistakes at times” (48-year-old participant). For some, acceptance was qualified with the assumption that the realities of the pandemic and lockdown were temporary. For others, acceptance was related to perseverance, expressed in living every day as it comes, or living life as usual; instead of focusing on the virus, they accepted its existence but also their agency in deciding how to live within the context of the pandemic. Lastly, some healthcare workers used more engaged acceptance, recognizing the existence of negative events and their individual agency to act within these challenges. They drew upon internal resources to understand their pandemic contexts and assess the resources available to best address it: “The mid phase [of the pandemic] gave other levels of staff the opportunity to accept and adapt to a new way of being with regards to hygiene protocols” (43-year-old participant). Healthcare workers adapted to the new reality of the pandemic by changing routines, such as limiting shopping trips, eating lunch outside, scheduling meetings where social distancing can occur, reading bibles when church services were cancelled, and using home equipment in lieu of going to the gym.

#### Manifestations of agency: Problem-solving and taking action

Adaptive acceptance of reality does not imply helplessness, and thread through many of the narratives were expressions of agency and examples of healthcare workers taking charge. Many participants chose to be a part of work committees supporting colleagues and developing solutions to the pandemic: “The planning team… also set up support groups–all voluntary. They held general support meetings, socially distanced, about COVID–educational videos, asking questions. The allied workers would also apply their skills to independently provide additional support–e.g., psychologists would run support groups, OT made masks using sewing machines to patients and staff and also ran educational events for COVID–hygiene, mask wearing, social distancing” (43-year-old participant). Healthcare workers also initiated groups to offer emotional or spiritual support to colleagues: “A special WhatsApp group for staff members who tested positive…served to provide support and encouragement, as well as information and answers to questions, for those in isolation. It appears that the staff on this group have been finding it very useful and helpful” (48-year-old participant).

#### Distraction: Refocused attention

Participants described refocusing their attention through engagement with hobbies, taking space from work, relaxing, and supporting others: “There were lots of other challenges… these [challenges] helped me realize that I need to focus and be a responsible citizen to empower people to be in a different situation, because this can benefit other people and give them the tools. So I strongly believe in mentoring others so they can be in a better situation. I get fulfilled when I have contributed to help someone grow” (51-year-old participant). Some participants exercised during the pandemic and lockdown, while others relied on movies and television to take a break from the stresses of work and life. Student participants mentioned refocusing on their studies. A small number of participants decided to stop watching and listening to the news as they found its contents, especially regarding COVID-19, too stressful.

#### Interpersonal coping

Interpersonal interaction was an important strategy for most participants and was predominately sought through varied social support systems. Participants living with family continued to meet in person, while social media groups became were helpful for keeping in contact with extended family and friends. Protocol and guideline documents and support groups were a common form of communication in hospitals. Social support relieved stress resulting from a positive COVID-19 diagnosis or illness, from work, and from the government lockdown. These interactions provided emotional support, increased confidence, and positive framing. Conversely, the erosion of social support systems resulting from the government lockdown was considered a challenge that increased the stress already felt during the pandemic. Many participants recognized more than one interpersonal support system in navigating pandemic and stress related to government restrictions, including friends and family, work colleagues, and work management.

Most healthcare workers turned to loved ones for support: “It was mostly like through the phone–talking to the friends and the family because I definitely could not see them…we were supporting each other through the phone. I would make sure that I would call them and check on them and give them support” (50-year-old participant). Relationships with husbands, siblings, co-workers, neighbors, and romantic partners were common sources of comfort, though therapy was also mentioned by a small number of participants. Family activities became even more important during this time, and exercising, sharing about their days, or simply talking could remove some of the isolation and stress that participants experienced. Other participants found solutions around the quarantine like phoning more often, with a minority of participants feeling that this brought their families closer together. For some participants who became ill, quarantining with exposed family members was a source of comfort, decreasing feelings of isolation. Participants also found family, friends, and colleagues to be helpful while they were ill, delivering goods like groceries when they were unable to go shopping themselves. It is important to note, though, that many participants also found living with family a source of stress because they feared putting loved ones at risk due to their illness or job.

Support from colleagues was also beneficial. Co-workers initiated support groups, spiritual wellness groups, or collaborative groups, to cope with both work stress and the pandemic more generally. Welcoming committees were established for co-workers who returned after recovering from COVID-19. Social media support groups were initiated. Colleagues phoned sick co-workers to show support. Shared experiences fostered a sense of comradery which decreased feelings of isolation, provided a place to debrief, and buffered against the stigma that some experienced because of work or illness. Healthcare workers would find support in informal conversations with colleagues or by swapping or covering shifts for those who were burned out, or rearranging schedules based on transportation challenges.

Collaborating work groups were also established, which functioned as a form of adaptive coping. Steering committees formed to brainstorm and find solutions to the current reality. These groups allowed colleagues to work together to pragmatically solve problems: “When there’s a crisis, there’s no drama because we put our heads together and figure it out, and also speak to people to get advice” (51-year-old participant). Participants felt that the groups increased confidence through knowledge provision and the sense of control felt by taking action in a stressful situation.

#### Structural coping

One additional form of social support participants used was structural; healthcare workers relied on guidance from institutional authorities. When hospitals were transparent, clear, and honest in their messaging, participants considered the communication as a generally a positive experience: “Good communication. Being transparent. Consultation of relevant stakeholders before decisions are made. These rather simple concepts were not always easy to achieve. But without them progress was difficult, and one would not get the necessary buy-in of all the role-players, leading to problems” (48-year-old participant). Good communication practices included consultation with relevant stakeholders and management collaborating with staff to create solutions to the pandemic. Hospitals were viewed as supportive when they provided additional training around the coronavirus, COVID-19, or the vaccine, and when they offered wellness resources. Finally, communication and collaborations between hospitals were viewed positively.

### Material coping

Participants used material resources to protect or support themselves physically, mentally, and emotionally, at home and at work, though some everyday concerns worsened during the pandemic.

Participants attempted to keep healthy by using vitamins, minerals, herbal remedies such as *umhlonyane* (or African wormwood), and traditional healing practices including ginger, garlic, eucalyptus, and steaming. A few participants who fell ill attempted to relieve symptoms by using flu or allergy medicine, painkillers, or antibiotics. Combinations of practices were also mentioned: “I was drinking umhlonyane, taking vitamin C, and Allergex” (30-year-old participant).

COVID-specific protections, including masks, sanitizer, and household cleaners, were also important. Some participants regularly cleaned groceries after purchase or bathed before entering their homes from work. Vaccine development was a source of comfort for some participants, “We won’t get the COVID if you take the vaccine and then, you know, you won’t infect other people as well, around you, in your household, you know” (an older participant, age unknown). This comfort, though, was tempered with a concern for the safety and effectiveness of the vaccines in development. COVID-19 testing and PPE were a source of comfort in the instances when it was available. For a minority of participants, the lockdown protocols were comforting, though most participants found them challenging.

Many participants found masks and mask requirements an important method for reducing transmission; in fact, masks were mentioned more frequently than any other COVID protection measure: “You must always wear a mask and to keep social distance” (44-year-old participant). Community members who were not following the rules became a source of stress. Social distancing and restricting movement, particularly in the hospital, was comforting to some participants, who then began to rely on the outdoors as both a method to social distance and a source of emotional and mental support. Finally, use of glass or plastic barriers and thermometers increased sense of safety for some.

Technology became a key resource, and social media groups flourished among healthcare workers both as socioemotional support systems and for communication purposes for healthcare workers: “We had a spiritual wellness group that I was coordinating at work. We used to send the weekly recording to the group, uplift them and then at home over the week also, we would connect and stream online services” (45-year-old nurse). Participants used these groups to keep in touch with family and friends, to show support for colleagues who were ill, or as a means of expressing solidarity. Social media groups provided a mode of communication at work, becoming one method for giving or receiving advice or updating guidelines or protocols. Hospitals also used informational emails and online meeting platforms to connect healthcare workers and management. Online meeting platforms were also used to attend religious services or write exams. A few participants also sought teletherapy for psychological support or counseling.

Lastly, some common or everyday resources changed in valence. Though many participants worried about finances, a minority expressed gratitude for receiving financial assistance, data, or airtime, or for having savings or investments to use during the pandemic: “The university, they gave us free data so we can study from home” (37-year-old male participant). Groceries could be a source of stress when they were tied to finances or when healthcare workers became ill with COVID-19, had to isolate, and were unable to shop. The provision of food became a support, though, when healthcare workers were gifted food hampers at work or received groceries from friends or family while they were ill and could not shop.

## Discussion

These results describe the multiple and diverse coping strategies within individual, residential, and hospital systems at intrapersonal, interpersonal, and structural levels, which were used by healthcare workers during the first year of the COVID-19 pandemic. Our results largely support previous literature by drawing upon a diverse sample of hospital-based public psychiatric healthcare workers in South Africa to identify common forms of coping during the COVID-19 pandemic, including positive mindsets and reappraisal, social support systems, and communication. Additionally, our findings demonstrate the subjectivity of coping and the interconnectedness of coping strategies and systems used within our sample.

### Common coping mechanisms for healthcare workers

Related to previous findings of cognitive reappraisal, attitude, positive attitude, or positive framing [15, 16, 18, 26, 30], healthcare workers in this study used mindset and reappraisal as emotion-focused coping strategies [26] to promote optimism and hope. The use of positive mindset and reappraisal within our sample is particularly important, as both may influence selection of other coping strategies and therefore encourage stress optimization as opposed to maladaptive coping techniques [17, 23], which could be leveraged in the development of pandemic-focused interventions [24]. Additionally, making meaning is associated with lower levels of PTSD [22] and so can directly impact mental health in adverse situations. Acceptance [13, 27, 28], self-efficacy [13, 30] and refocusing attention to activities such as exercise or self-distraction [27, 33] have also been used as coping mechanisms by healthcare workers during this and previous pandemics. Refocused attention may be an effective form of emotional regulation [26] and self-efficacy is associated with greater problem-focused coping and job satisfactions [41], whereas acceptance–when used mindfully–may promote both problem-focused engagement and cognitive reappraisal [42, 43]. Interestingly, “deliberate rumination” reminiscent of the introspection mentioned by two participants was found to be associated with post-traumatic growth in one study [16]. It is possible that, to the extent that introspection as a form of self-observation and awareness is related to the process of mindfulness [40, 42], it may encourage problem-focused coping [42].

Social support was one of the most endorsed coping strategies [15], which is notable in the role that relationships may play in decreasing the risk of mortality [44] and mental health issues [45, 46]. Family support specifically has been linked with increased active coping [47] and decreased emotional exhaustion [48], perhaps providing an explanation for its importance in our sample. Support from families operated through multiple pathways and served different purposes, and the flexibility and adaptability displayed in seeking and maintaining relationships may be indicative of the importance of social support systems in times of stress [47]. Additionally, comradery fostered between co-workers provided both emotional support and functional adaptations such as sharing or swapping shifts, which are associated with improved wellbeing through decreased burnout [49] and mental health issues [50, 51], and increased job retention [19, 52]. Communication between colleagues provided interpersonal connection and knowledge, which is associated with increased confidence [7, 13–15, 19, 28, 30, 31]. Good communication and collaboration with management, described as being transparent, clear, and informative were also a source of support, possibly providing a buffer between traumatic stress at work and burnout or intent to resign [13, 51].

### Contextual and subjective nature of coping strategies

Participants disagreed on the value of some potential resources, which reflects the contextual and subjective nature of coping [17, 18]. Environmental demands [18] and access [53, 54] play a large role in determining whether a material resource mediates a coping or stress response [55]. Socioeconomic status affected coping by influencing whether participants felt stress, anxiety, and worry about financing groceries and transportation. Access similarly influenced perspectives on PPE, technology, and communication–when available and adequate they were supportive, but their absence resulted in stress.

The role of families were simultaneously supportive and stressful, and previous findings have suggested that families can be both sources of stress [48] and coping [47, 48] in nurses trying to achieve a work-life balance. Though families were generally discussed positively, the concerns felt by the participants for family members’ health and safety were serious and added nuance to the supportive nature of families, which itself was qualified by frustrations that lockdown was preventing in-person interaction. Participants who lived with their families had the comfort of in-person support but were also worried about transmitting COVID-19 from work to their families. Families could be a source of financial stress, as participants took in family members who had lost employment because of the government lockdowns. It appears that, similar to previous findings [48], family support provided more emotional coping during the pandemic than before, but that the pandemic also increased family-related stressors.

Some factors seemed variably supportive, depending upon how they were conceptualized [20, 56] and operationalized [57]. Acceptance has been found to be supportive in healthcare workers during the COVID-19 pandemic [13, 33] but the results of this study suggest that nuance may be warranted, as acceptance was practiced by many participants but with different manifestations. Some participants accepted the newness and consequent likelihood of errors during the pandemic, allowing for mistakes and implying the ability to correct early errors. In fact, some participants described an adaptive acceptance [43], where they worked to understand the situation, assess available resources, and determine best practices for managing outcomes due to the pandemic. Other participants described a passive acceptance [58] that the pandemic was the new reality and therefore expressed a desire to live life as usual, without focusing on the virus. In a minority of interviews, participants fatalistically accepted the inevitability of contracting the virus. This is in line with recent literature which suggests that acceptance may be misused, leading to maladaptive and counterintuitive suppression of emotions and denial of their impact [42]. The nature of passive or fatalistic coping is in opposition to the open reflection required for adaptive acceptance [40] and can be difficult to interpret. Van Breda [58] noted different levels of protective coping afforded through different types of acceptance within the South African context, suggesting that passive acceptance may be associated with helplessness and diminished agency or self-efficacy. Additionally, fatalism could discourage the use of protective factors such as masks, and future research should clarify the different understandings and uses of acceptance in relation to effective coping.

### Interconnectedness of coping mechanisms

This study suggests a high degree of interconnectedness between and within systems and levels of coping, while also demonstrating the flexibility of coping strategies through the integrated use of higher order adaptive processes [20]. These systems and processes do not operate in isolation and can function to either strengthen or weaken a global support network [59]. Most notable is the effect of work on participants’ intrapersonal attitudes during the pandemic, with some participants expressing gratitude for employment and others finding personal fulfillment and value in their work, which helped to buffer them against stigma. As mentioned above, these reappraisals and positive mindsets may be of particular interest as a central factor connecting other coping strategies [23], such that a positive mindset may in turn influence healthcare workers sense of agency or interpersonal interactions both at home and at work. Meaning making has also been found to be associated with active acceptance [42, 43, 60] which may be related to distraction [20], illustrating the complexity of coping networks. Additionally, strong teamwork and clear communication decreased stress by allowing individuals to express themselves while also providing planning, structure, and pandemic training, which in turn increased confidence facilitated work between colleagues. Future research should further explore these interactions between coping strategies, their impact on the coping network, and the association between these intra-network interactions and stress outcomes in order to inform the development of interventions to support healthcare workers.

## Conclusion

This study explored multilevel, multisystemic coping mechanisms used by healthcare workers employed in tertiary hospitals in Gauteng, South Africa. Most common systems of support included personal attributes, mindset, acceptance, agency, and reframing at the intrapersonal level; family and coworker support at the interpersonal level; and effective communication at the structural level. Additionally, technological, financial, medicinal, gustatory, and COVID-specific material resources were used across all levels. Coping mechanisms were similar to earlier findings, have nuance in their utilities, and interact with one another. This research extends previous literature on coping in healthcare workers during the COVID-19 pandemic and can be used to inform future interventions to support healthcare workers facing health crises.

## Supporting information

S1 File (DOCX)

S2 File (DOCX)
