# Supplementary material for: Coping strategies employed by public psychiatric healthcare workers during the COVID-19 pandemic in southern Gauteng, South Africa
Source: PLoS One. 2023 Aug 10;18(8):e0277392. doi: 10.1371/journal.pone.0277392 (PMC10414601; doi:10.1371/journal.pone.0277392)
Supplement: S1 File — (DOCX) [file pone.0277392.s001.docx]

**Interview Guide for PsyCOVID: Perceptions, Experiences, and Impacts of the COVID-19 Pandemic among Patients and Healthcare Workers in Tertiary Hospitals in Gauteng Province**

Part I. COVID-19 perceptions and experiences

1) To start, please tell me a little bit about yourself.

- Probe: family, number of children, hobbies, etc.

2) Describe your life before the pandemic started in the beginning of 2020.

- [open ended for the participant to explain their experience pre-pandemic, if more details required, can then probe employment, finances, family life, etc.]

3) What was your experience at the beginning of the coronavirus pandemic and lockdown (middle to end of March)?

- [open ended for the participant to explain their experience, if more details required, can then probe employment, finances, family life, etc.]

4) What has been challenging during the pandemic? How has this changed overtime?

- Probe: mental health, finances, an infected loved one

5) Did you ever “think too much”/excessively ruminate? If so, about what? How does it affect you?

What has helped you get through the pandemic?

- Probe: social supports, coping mechanisms, financial assistance, faith, herbal remedies

Part II. COVID-19 clinical experiences and conditions among healthcare workers

*Personal experiences and risk*

1) When and how did you first hear about COVID? What were your thoughts and how did you feel?

2) After the Level 5 lockdown began in late March, how did the lockdown affect you and your work in the psychiatry ward? How did this change over the course of the lockdown?

3) After Level 5 began, how did the pandemic affect you personally? How did this change over the course of the lockdown?

Probe: mood, emotional well-being, mental health, physical health, finances

[If no mention of mental health] How do you think the pandemic affected your mental health?

4) How did the lockdown affect your family? How did this change over the course of the lockdown?

Probe: finances, food, stress, fighting, feeling closer with each other, etc.

5) Were there any moments during the pandemic that you felt unsafe at work?

Probe: infection, PPE, admitting new patients, transport, work relationships

6) Did you ever feel burnt out at work before or during the pandemic?

7) How did you balance your work priorities, home priorities, and other life priorities during COVID?

*Positive Coping and Support*

1) What has helped you successfully cope during the pandemic?

- Probe: family, friends, religion, drugs, going to work, etc.

2) How are you receiving social support during the pandemic?

- Probe: family, neighbours, seeing people in-person, videochatting, online church, going to work

3) Is it difficult to receive social support? Why or why not?

- Probe: internet access, cost of cellphone minutes, services offered (e.g. church services)

4) What resources would have been helpful for healthcare workers during the worst of the pandemic?

5) What resources will be helpful for healthcare workers moving forward?
