# Supplementary material for: Coping strategies employed by public psychiatric healthcare workers during the COVID-19 pandemic in southern Gauteng, South Africa
Source: PLoS One. 2023 Aug 10;18(8):e0277392. doi: 10.1371/journal.pone.0277392 (PMC10414601; doi:10.1371/journal.pone.0277392)
Supplement: S2 File — (DOCX) [file pone.0277392.s002.docx]

**CODEBOOK**

**Coping and stressors during the COVID-19 pandemic among patients and healthcare workers in Tertiary Hospitals in southern Gauteng Province.**

Table of Contents

1. Individual Coping
2. Interpersonal Coping
3. Material Coping

**1.** **INDIVIDUAL COPING**

***Coping mechanisms:*** any form of coping methods mentioned (e.g. acceptance, trusting in God, Church/religion, using natural remedies, etc.)

***Self-care***: any mention of self-care practices and the importance of it (e.g. taking medication, bathing, wearing mask, etc.)

***Future orientation****:* any reference to goals, forward thinking, hope for the future, or other indication of optimism and hopeful thinking about the future

***Appreciation:*** any mention of positive recognition of or gratitude for a particular thing/situation/action

***Agency:*** any reference to problem-solving or agency, indicating that the participant feels they have the ability to act in a way that will produce a positive change

***Hobbies:*** any mention of something done for fun or to relax

***Change (Acceptance):*** any expression indicating acquiescence, letting-go of a situation, or assenting to a situation without protest

***Change (Adaptation):*** any expression of changes adopted as a consequence of the new reality of COVID

***Change (Distraction/Refocused Attention):*** any expression of actively seeking a new focus for attention, beyond the stressors induced by COVID

**2. INTERPERSONAL COPING**

***Family Social Support:*** any mention of supportive family members (including spouse, children or extended family members.)

***Coworker Social support/ Supportive staff:*** any mention of receiving support from co-workers

***Virtual Social Support:*** any form of virtual support (e.g. online sermons, video chats with friends and family etc.)

***Friend Social Support:*** any mention of receiving support from friends

***Management Social Support:*** any mention of receiving support from management at work

***Effective communication:*** any mention of management’s communication style being sufficient (e.g management did the best they can.)

***Communication style:*** any mention of any communication style used by management (bulletins, meetings, etc.)

**3. MATERIAL COPING**

***Herbal remedies:*** any mention of any herbal/home remedies to prevent/cure COVID-19

***Vaccine:*** any mention of vaccine to prevent COVID-19

***Infrastructure/resource development:*** any reference to the development of new resources or infrastructure to help cope

***Resource assistance:*** any reference to friends/family/institutions providing established resources (wifi, food, etc) to help the participant cope
